# Clinical usefulness of capnographic monitoring when inserting a feeding tube in critically ill patients: retrospective cohort study

**DOI:** 10.1186/s12871-016-0287-x

**Published:** 2016-12-09

**Authors:** Jeong-Am Ryu, Kyoungjin Choi, Jeong Hoon Yang, Dae-Sang Lee, Gee Young Suh, Kyeongman Jeon, Joongbum Cho, Chi Ryang Chung, Insuk Sohn, Kiyoun Kim, Chi-Min Park

**Affiliations:** 1 Department of Critical Care Medicine, Samsung Medical Center, Sungkyunkwan University School of Medicine, Seoul, Republic of Korea; 2 Department of Surgery, Samsung Medical Center, Sungkyunkwan University School of Medicine, Seoul, Republic of Korea; 3 Division of Cardiology, Department of Medicine, Samsung Medical Center, Sungkyunkwan University School of Medicine, Seoul, Republic of Korea; 4 Division of Pulmonary and Critical Care Medicine, Department of Medicine, Samsung Medical Center, Sungkyunkwan University School of Medicine, Seoul, Korea; 5 Research Institute for Future Medicine, Biostatistics and Clinical Epidemiology Center, Samsung Medical Center, Seoul, Korea

**Keywords:** Capnographic monitoring, Feeding tube, Critically ill patient, Pneumothorax

## Abstract

**Background:**

It is not rare for a small-bore feeding tube to be inserted incorrectly into the respiratory system in critically ill patients. Thus, monitoring is necessary to prevent respiratory malplacement of the tube. We investigated the utility of capnographic monitoring to prevent respiratory complications due to feeding tube mispositioning in critically ill patients.

**Methods:**

This study was a pre and post-interventional study, including 445 feeding tube placements events studied retrospectively in the medical and surgical intensive care units of the Samsung Medical Center. We compared outcomes between time periods before and after capnographic monitoring and documented any respiratory complications.

**Results:**

Feeding tubes were inserted in 275 cases without capnographic monitoring. Capnographic monitoring was performed in 170 cases. Sixteen patients (4%) had respiratory complications of all tube placements. Feeding tube was inserted into the trachea in 11 (2%) patients and for a pneumothorax in five (1%) patients. Fourteen cases of respiratory complications were detected in the control group (14/275, 5%, 10 tracheal insertions and four pneumothoraxes). Two respiratory complications were detected in the capnographic monitoring group (2/170, 1%, one tracheal insertion and one pneumothorax). Respiratory complications were detected less frequently in the capnographic monitoring group than that in the control group (*P* = 0.035).

**Conclusions:**

Capnographic monitoring is simple, easy to learn, and may be useful to prevent respiratory complications when placing a feeding tube in a critically ill patient.

## Background

Small-bore feeding tubes have been used to provide proper nutrition and deliver medications to critically ill patients. Inserting a feeding tube is a routine procedure in many intensive care units (ICUs). However, complications associated with inserting a feeding tube have been reported. A major complication of inserting a feeding tube is accidental placement of the tube in the respiratory system, resulting in pneumothorax, hydropneumothorax, bronchopleural fistula, atelectasis, empyema, pneumonitis, or pneumonia. These complications are associated with significant morbidity and mortality [1–4]. However, it is not rare for a small-bore feeding tube to be inserted incorrectly into the respiratory system [2, 5–8].

Critically ill patients with an endotracheal tube and tracheotomy may exhibit poor response due to their sedated state. Use of an assistant guidewire may contribute to respiratory complications when inserting a feeding tube [9–11]. These conditions are thought to be potential risk factors for tracheobronchial malpositioning of a feeding tube in a critically ill patient [2, 9–11].

Several methods have been proposed to prevent tracheal malpositioning of a feeding tube. However, some of the methods are not effective, not cost-effective, or require a specialist to prevent respiratory complications when inserting a feeding tube [6, 7, 9]. Capnographic monitoring may be a more effective to prevent respiratory malpositioning of a feeding tube and is a simple and cost-effective method [3, 6, 12–15]. Several studies have reported that capnography helps to prevent respiratory tract malpositioning and confirm feeding tube position. However, these studies were small pilot studies or demonstrated only fragmentary evidence to support its usefulness [3, 12, 14, 15]. We investigated the usefulness of capnographic monitoring to prevent respiratory complications when inserting a feeding tube in a critically ill patient.

## Methods

This retrospective study was performed in a cohort of patients who underwent insertion of a small-bore feeding tube during hospitalization at the medical or surgical ICUs of Samsung Medical Center (1961-bed, university-affiliated, tertiary referral hospital in Seoul, South Korea) from August 2014 to January 2015. This study was approved by the Institutional Review Board of Samsung Medical Center (SMC 2015-02-076-001) according to the Declaration of Helsinki on reviewing and publishing information from patient’s records. Informed consent was waived because of the retrospective nature of the study.

### Patients

A total of 445 feeding tube placements were studied retrospectively. Eligible subjects were > 18-years-of-age and required a feeding tube according to the ICU physician during their hospital stay. Capnographic monitoring was performed to prevent respiratory tract malplacement while inserting feeding tubes from December 2014 to January 2015. We compared time periods before and after implementing the capnographic monitoring technique. Capnographic monitoring was not performed when tubes were used from August 2014 to October 2014. We defined the feeding tube placements during this period as the control group. Patients were excluded if feeding tube insertion failed or a chest X-ray was not performed to confirm feeding tube position.

### Data collection

The feeding tubes used in all cases were small-bore, flexible feeding tubes (Covidien Kangaroo, Entriflex™ Nasogastric Feeding Tubes, 12 Fr, 60 in; Covidien, Dublin, Ireland) and an assistant guidewire was used. A chest X-ray was always performed to confirm the position of the feeding tube. We defined respiratory complications as feeding tubes that were inserted into the tracheopulmonary system (respiratory malplacement of the tube) or direct/indirect lung injuries during an attempt to insert a feeding tube with complications such as pneumothorax, hydropneumothorax, bronchopleural fistula, atelectasis, empyema, pneumonitis, and pneumonia. An artificial airway was defined as having an endotracheal or tracheal tube in place while the feeding tube was being inserted. The Glasgow Coma Scale (GCS) and the Richmond Agitation Sedation Scale (RASS) were used to check patients’ mental status and level of sedation. Delirium was defined when the confusion assessment method for the ICU (CAM-ICU) was positive. All variables were investigated when feeding tubes were inserted, except reason for the ICU admission.

### Capnographic monitoring protocol

Each feeding tube was placed according to the following protocol. Tube insertion was monitored with a capnographic unit (Covidien, Microcap® Handheld Capnograph). The capnography unit was calibrated according to the manufacturer’s instructions. The capnographic unit was turned on and connected to the side hole of the feeding tube. Carbon dioxide (CO_2_) level and flow waveforms were monitored continuously during tube insertion. Placement within the airway was defined as detecting a capnogram wave or an end-tidal carbon dioxide (ETCO_2_) level > 5 mm Hg. The feeding tube was inserted via a nasal approach to distance of 30 cm and then held at the tip of the monitor guidewire to detect CO_2_ for > 20 s (first step). The tube insertion procedure was stopped if a CO_2_ waveform appeared or ETCO_2_ level was > 5 mm Hg. Then, the feeding tube was removed and reinsertion was attempted. If ETCO_2_ was not detected during the first step, the feeding tube was inserted the planned distance, and the physician made certain that ETCO_2_ was monitored for an additional 20 s (second step). If the airway became involved, the feeding tube was removed by the physician and retried. Finally, a chest X-ray was performed to confirm feeding tube position.

### Statistical analyses

Data are presented as medians and interquartile ranges (IQRs) for continuous variables and as numbers (percentages) for categorical variables. The data were compared using Mann-Whitney U test for continuous variables and the chi-square or Fisher’s exact tests for categorical variables. All tests were two-sided, and *P* < 0.05 was considered significant. Data were analyzed using IBM SPSS Statistics 20 software (IBM, Armonk, NY, USA).

## Results

A total of 445 feeding tube placements that took place in either the medical or surgical ICUs were analyzed. Of them, 275 cases (August–October 2014) did not have capnographic monitoring performed during insertion. Capnographic monitoring was implemented in December 2014, and 170 feeding tube insertion cases used capnographic monitoring. The median age of the patients was 67 (range 56–75) years, and 294 patients (66%) were male. The most common reason for ICU admission was respiratory failure. The baseline characteristics of the capnographic monitoring and control groups are shown in Table 1. There were a few differences between the two groups. Major reasons for ICU admission and ICU locations were different between the two groups. Mechanical ventilator use was more prevalent in the capnographic monitoring group than that in the control group (*P* < 0.001). GCS was higher in the capnographic monitoring group than that in the control group (*P* = 0.005). Sixteen patients (4%) had respiratory complications among all tube placements. Tracheal insertion occurred in 11 (2%) patients and pneumothorax in five (1%). No complication-induced death occurred in either group. Fourteen control group cases had respiratory complications (14/275, 5%, 10 tracheal insertions and four pneumothoraxes). Two cases of respiratory complications occurred in the capnographic monitoring group (2/170, 1%, one tracheal insertion and one pneumothorax). Elevated ETCO_2_ level or capnogram wave was detected in 12 cases in the monitored group (12/170, 7%), and lung injury (pneumothorax) was reported in only one case. False negative capnographic monitoring occurred in 1% of cases (tracheal insertion of feeding tube, but CO_2_ was not detected during one tube placements). No differences were in the demographics detected between the groups with and without respiratory complications in a univariate analysis, except sex (Table 2). More respiratory complications occurred in male patients. Respiratory complications were detected less frequently in the capnographic monitoring group than that in the control group (*P* = 0.035). Respiratory complications were not associated with age, level of consciousness, mechanical ventilator use, artificial airway, or use of a sedative drug in this study (Table 2).

**Table 1:** Baseline characteristics of the capnographic monitoring and control groups

	Overall (*n* = 445)	Capnographic monitoring group (*n* = 170)	Control group (*n* = 275)	*P*-value
Age (years)	67 (56–75)	68 (59–75.75)	64 (55–75)	0.221
Gender (male)	294 (66)	109 (64)	185 (67)	0.537
Major reasons for ICU admission				
Respiratory failure	209 (47)	81 (48)	128 (47)	0.002
Cardiovascular	72 (16)	17 (10)	55 (20)	
Severe sepsis or septic shock	81 (18)	45 (26)	36 (13)	
Neurological	49 (11)	15 (9)	34 (12)	
Post-operation	17 (4)	4 (2)	13 (5)	
Multiple trauma	8 (2)	3 (2)	5 (2)	
Other	9 (2)	5 (3)	4 (1)	
Use of mechanical ventilator	332 (75)	143 (84)	189 (69)	<0.001
Artificial airway	366 (82)	151 (89)	215 (79)	0.004
Endotracheal tube	242 (54)	103 (61)	139 (51)	
Tracheal tube	124 (28)	48 (28)	76 (28)	
Location				
MICU	165 (37)	62 (36)	103 (38)	0.031
SICU	12 (3)	5 (3)	7 (3)	
CCU	46 (10)	10 (6)	36 (13)	
Cardiac SICU	11 (2)	2 (1)	9 (3)	
Oncology MICU	118 (27)	45 (26)	73 (27)	
Oncology SICU	93 (21)	46 (27)	47 (17)	
GCS	13 (11–15)	14 (12–15)	13 (10–15)	0.005
RASS	0 (−2–1)	0 (−2–1)	−1 (−2–1)	0.717
Delirium	203 (59)	86 (63)	117 (57)	0.216
Use of sedative drug	207 (47)	88 (52)	119 (43)	0.096
Respiratory complication	16 (4)	2 (1)	14 (5)	0.031
Tracheal insertion	11 (3)	1 (1)	10 (4)	
Pneumothorax	5 (1)	1 (1)	4 (2)	

*ICU* intensive care unit, *MICU* medical intensive care unit, *SICU* surgical intensive care unit, *CCU* cardiac intensive care unit, *GCS* Glasgow Coma Scale, *RASS* Richmond Agitation Sedation Scale

**Table 2:** Comparisons between the respiratory complication and non-complication groups

	Overall (*n* = 445)	Respiratory complication group (*n* = 16)	Non-complication group (*n* = 429)	*P*-value
Age (years)	67 (56–75)	69 (53–80)	67 (56–75)	0.669
Gender (male)	294 (66)	15 (94)	279 (65)	0.015
Major reasons for ICU admission				
Respiratory failure	209 (47)	9 (56)	200 (47)	0.493
Cardiovascular	72 (16)	3 (19)	69 (16)	
Severe sepsis or septic shock	81 (18)	1 (6)	80 (19)	
Neurological	49 (11)	1 (6)	48 (11)	
Post-operation	17 (4)	1 (6)	16 (4)	
Multiple trauma	8 (2)	0 (0)	8 (2)	
Other	9 (2)	1 (6)	8 (2)	
Use of mechanical ventilator	332 (75)	14 (88)	318 (74)	0.379
Artificial airway	366 (82)	15 (94)	351 (82)	0.326
Endotracheal tube	250 (55)	111 (60)	139 (51)	
Tracheal tube	129 (28)	53 (29)	76 (28)	
Location				
MICU	165 (37)	8 (50)	157 (37)	0.706
SICU	12 (3)	1 (6)	11 (3)	
CCU	46 (10)	1 (6)	45 (10)	
Cardiac SICU	11 (2)	0 (0)	11 (3)	
Oncology MICU	118 (27)	3 (19)	115 (27)	
Oncology SICU	93 (21)	3 (19)	90 (21)	
GCS	13 (11–15)	14 (12–15)	13 (11–15)	0.915
RASS	0 (−2–1)	1 (−1–1.3)	0 (−2–1)	0.153
Delirium	203 (59)	8 (62)	195 (59)	0.860
Use of sedative drug	207 (47)	10 (63)	197 (46)	0.211
Two-step technique	102 (23)	6 (38)	96 (22)	0.220
Use of capnography	170 (38)	2 (13)	168 (39)	0.035

*ICU* intensive care unit, *MICU* medical intensive care unit, *SICU* surgical intensive care unit, *CCU* cardiac intensive care unit, *GCS* Glasgow Coma Scale, *RASS* Richmond Agitation Sedation Scale

## Discussion

We evaluated the usefulness of capnographic monitoring for preventing respiratory complications and the clinical characteristics associated with inserting a feeding tube in a critically ill patient. The results indicate that capnographic monitoring was useful to prevent respiratory complications while inserting a feeding tube in a critically ill patient. Respiratory complications were not associated with altered mental status, artificial airway, or use of sedative drugs in this study. Respiratory complication rates in all-comers were 4% in this study. Thus, it is not rare for a small-bore feeding tube to be inserted incorrectly into the respiratory system in a critically ill patient.

Malplacement of a feeding tube in the respiratory tract can lead to various complications associated with significant morbidity and mortality [9]. Several studies have reported that small-bore feeding tubes are inserted into the tracheopulmonary system in 0.3–15.0% of placement attempts [2, 5–8]. Overall malpositioning-induced complications, such as pneumothorax and hemothorax, occur (0.7–1.2%) and complication-induced death in 0.3% of cases [2, 6, 7]. Most of the patients who developed complications used a mechanical ventilator (14/16, 88%) and an artificial airway (15/16, 94%) in our study. Assistant guidewires were used in both groups. Decreased or delirious mentality is a risk factor for respiratory malpositioning of a feeding tube [2, 16, 17]. Sedative and analgesic agents are used widely in critically ill patients because of pain, delirium, and agitation. Critically ill patients using a mechanical ventilator may exhibit a poor response due to sedation. These conditions are thought to be potential risk factors for tracheobronchial malpositioning of a feeding tube [2, 10, 11]. An artificial airway is actually a mechanical barrier; however, the low pressure cuffs of endotracheal and tracheal tubes do not represent a reliable barrier [1, 11]. If an assistant guidewire is used, the small, flexible tube may slide into the trachea by the cuff along the tracheal wall [10]. Therefore, critically ill patients may be vulnerable to respiratory complications when inserting a tube.

Conventional methods of confirming tube position include auscultation of a bubbling sound in the epigastric region during air insufflation and aspiration of gastric fluid. However, these conventional methods are not useful and accurate to identify a malpositioned feeding tube [2–4]. Therefore, alternatively methods are required to verify feeding tube placement and prevent respiratory system complications. Chest X-ray is the gold standard to detect tracheal tube malpositioning [9, 11]. This method confirms position after inserting the tube, but it is impossible to prevent respiratory malpositioning [9]. Thus, other methods to prevent a tube from entering the respiratory system are needed. Several approaches have been developed, such as fluoroscopic-, laryngoscopic-, and endoscopic-guided insertion. However, these techniques increase cost and time and require a specialist [2, 7]. Capnographic monitoring is a simple, cost-effective, and easily implemented. In addition, no specialist is needed for capnographic monitoring. Recent studies have reported advantages of bedside electromagnetic (EM)-guided feeding tube placement by specialized nurses [18–20]. Although EM-guided tube placement may help prevent respiratory complications while inserting a feeding tube, capnographic monitoring is more cost-effective and simpler. Capnographic monitoring is useful to prevent respiratory malpositioning while inserting a feeding tube [3, 6, 12, 15, 21].

Two respiratory complications cases were detected in the capnographic monitoring group. One patient was obese and was on a mechanical ventilator, making it difficult to insert a tube after repeated attempts. Another patient had used sedative drugs and mechanical ventilator. Tube insertion was difficult and subsequent attempts led to a pneumothorax. CO_2_ was repeatedly detected during capnographic monitoring but the respiratory complication could not be prevented.

This study had several limitations. This was a retrospective review of medical records at a single institution. A positive capnogram may not truly reflect malplacement of a feeding tube. No method was available to confirm false positives during capnographic monitoring. Delirium was defined when CAM-ICU was positive in this study. CAM-ICU is an objective and useful screening test to discriminate delirium. However, CAM-ICU cannot be performed when the patient is irritable or in deep sedative state (RASS ≤ 3). Sedative drugs were used when mechanical ventilator was applied or delirium was observed. Although sedative drugs were used when feeding tubes were inserted, adequate sedation may not have occurred in some patients. No comparison was made between times to place the feeding tubes between the groups. The individual characteristics and the skill of the physicians inserting the feeding tubes were not considered.

## Conclusions

It is not rare for a small-bore feeding tube to be inserted incorrectly into the respiratory system in a critically ill patient. Respiratory complications decreased remarkably when capnographic monitoring was used while inserting a feeding tube. In addition, capnographic monitoring is a simple and easy to learn procedure, and no specialist is required. Our results reveal that capnographic monitoring is a simple and useful method to prevent respiratory complications while inserting a feeding tube in a critically ill patient.

## Abbreviations

ARDS: Acute respiratory distress syndrome
CAM-ICU: Confusion assessment method for the intensive care unit
CO_2_: Carbon dioxide
EM: Electromagnetic
ETCO_2_: End tidal carbon dioxide
GCS: Glasgow Coma Scale
ICU: Intensive care unit
IQR: Interquartile range
RASS: Richmond Agitation Sedation Scale
